# Facility and patient barriers in the implementation of isoniazid preventive therapy for people living with HIV attending Care and Treatment Centers, Songea Municipality, Tanzania

**DOI:** 10.11604/pamj.2021.38.197.26752

**Published:** 2021-02-22

**Authors:** Festo Faustine Komba, Gasto Frumence

**Affiliations:** 1 USAID, Sustaining Health Outcomes through the Private Sector (SHOPS) Plus Project, Dar es Salam, Tanzania,; 2 Department of Development Studies, School of Public Health and Social Sciences, Muhimbili University of Health and Allied Sciences, Dar es Salam, Tanzania

**Keywords:** Isoniazid preventive therapy, barriers, IPT coverage, Tanzania

## Abstract

**Introduction:**

isoniazid preventive therapy for people living with HIV is an essential public health intervention in low-income countries with high tuberculosis and HIV burden. Despite available evidence that it is efficacious, its implementation is still low in many countries. This study was designed to determine its implementation coverage and explore barriers for suboptimal implementation in Songea municipality in Tanzania.

**Methods:**

a cross-sectional descriptive study design using both quantitative and qualitative approaches of data collection was employed. A review of 2148 records of people living with HIV eligible for isoniazid preventive therapy (IPT) was done to determine its implementation coverage. Twenty-one (21) in-depth interviews and 5 observations were conducted to explore barriers in the implementation. Quantitative data was analyzed using Statistical Package for the Social Science (SPSS) for windows version 20 statistical software. Descriptive statistics (frequencies and percentage) were employed and data were visualized using tables and bar graphs. All interviews were audio-recorded and analyzed using thematic analysis approach.

**Results:**

overall, isoniazid preventive therapy coverage at Songea municipality was estimated to be 45%. Insufficient drug supply and stock out, shortage of staff, lack of service privacy, long waiting time, drug side effects, pills burden, distance and cost of transport were the main reported barriers hindering full scale implementation of isoniazid preventive therapy.

**Conclusion:**

implementation of isoniazid preventive therapy in Songea municipality had low coverage. The study recommends that tuberculosis and HIV stakeholders must be part of the solutions by ensuring that the identified barriers are addressed.

## Introduction

Isoniazid Preventive Therapy (IPT) for people living with HIV/AIDS (PLHIV) is crucial and essential for public health interventions in low-income countries with high TB and HIV burden. IPT is anti-tuberculosis drug provided to individuals who are latently infected with *Mycobacterium tuberculosis* in order to prevent the progression to active disease [1,2]. HIV is the most powerful known risk factor for progression from latent infection with *Mycobacterium tuberculosis* to active disease [2]. IPT is part of the three I´s for HIV/TB collaboration [2]. These include, Intensified TB case finding, Isoniazid (INH) preventive therapy and Infection control for TB in health care and congregate settings [2-4]. According to the World Health Organization (WHO), Tanzania is one of the 22 high burden countries for tuberculosis (TB) [5,6]. In Tanzania IPT was included in national policy guideline for collaborative TB/HIV activities in 2008 [7]. The 2015 national HIV guideline stipulate that all PLHIV should be screened for active TB using WHO screening algorithm that include four clinical symptoms (current cough, fever, weight loss and night sweats) [8]. For those whom active TB have been excluded IPT is administered daily, orally for a period of six months at a dose of 300 mg for adults and 10 mg/kg for children [1,4]. When IPT is provided to an eligible PLHIV, it provides up to 18 months of protection against TB [8]. IPT can be initiated before or after the initiation of anti-retroviral therapy [4,8,9].

The benefit of IPT among PLHIV have been demonstrated by different studies [8,10]. A Cochrane review of 12 randomized controlled trials revealed that IPT reduces the risk of developing active TB in PLHIV by 33% and by 64% when targeted to PLHIV who had a positive tuberculin skin test (TST) [4,10]. A retrospective study done in Rio Brazil 2007 also shows that IPT significantly reduces the incidence of TB among PLHIV who received Anti-Retroviral Therapy (ART). Tuberculosis incidence among patients who received ART and IPT was 0.80/100 PY (95% CI 0.38-1.47) [11]. Despite available evidence that IPT is efficacious and the existence of the WHO recommendation since 1998, its implementation is still low in many countries [6]. Globally, in 2014, a total of 933,000 PLHIV were provided with IPT, which is an increase from 600,000 people in a previous year. Of note is that, 77% of the countries in the world did not report provision of IPT as part of HIV care in 2014 [6,12]. Moreover, in Africa a total of 875, 886 PLHIV were provided with IPT in 2014. In Tanzania, only 23,124 PLHIV provided with IPT in 2014 representing 2% only [6]. Amongst reasons that have been pointed out for the low implementation of IPT globally are lack of social support, stigma, distance from the health care facility, pills burden, and drug side effects, lack of IPT training, lack of supportive supervision, health care providers´ attitude towards IPT (fear of isoniazid resistance), lack of good client-provider communication, and lack of patient registers [12-15]. In Tanzania, limited evidence is available regarding the coverage and barriers in the implementation of IPT for PLHIV. Therefore, this study aimed to reduce such knowledge gap by determining its implementation coverage and exploring barriers for IPT implementation in care and treatment centers (CTCs) in Songea municipality in Tanzania.

## Methods

The study was conducted in Songea municipality in Ruvuma region in the southern part of Tanzania. Songea municipality is among the districts of Ruvuma region and is the capital town of the region. As of 2012 population census, Songea municipality had a total population of 203,309 people (96,347 males and 106,962 females). This study setting was selected because Songea municipality was among the districts with high prevalence of HIV (5.6%) in Ruvuma region. This prevalence was higher than the national prevalence of 5.0% [16]. Songea municipality has 11 health care facilities providing IPT for PLHIV. Among these health care facilities eight are public facilities, two are military facilities and one is faith-based health facility. A cross-sectional descriptive study design using both quantitative and qualitative approaches of data collection was employed. Cross-sectional studies form a class of research methods that involve data collection at one specific point in time. This type of study design allows for quick and easy data collection even for a small or large population. The quantitative part aimed to determine IPT coverage of the municipality while the qualitative part aimed to explore facility and patient barriers in the implementation of IPT for PLHIV in CTCs in Songea municipality.

Purposive sampling technique was used to get study participants in both quantitative and qualitative part. The study participants for quantitative part included all PLHIV registered in CTCs of the selected health care facilities. Convenience sampling technique was used to select five out of 11 health care facilities providing IPT in the municipality based on their proximity, time allocated for data collection and the resource available for the research. The study participants for qualitative were also purposively selected because of their role in the implementation of IPT. They included health care providers namely ART-nurse and counselors, CTC clinicians and pharmacists who were working in the five selected CTCs. In addition, adults PLHIV who were provided with IPT in the five selected CTCs in Songea municipality were also included in the study. A total of 13 health care providers and 8 adults PLHIV provided IPT on CTCs of the five selected health care facilities participated in this study. The study participants were identified and approached with the help of the CTC in-charge of each health care facility selected. The number of health care providers and PLHIV interviewed depended on information saturation. In this study data saturation was reached when no more new information that was being generated, and when further coding was no longer feasible [17]. In-depth interviews were conducted in each selected health care facility using well designed in-depth interview guides. An observation checklist was also used to collect qualitative information. Observation checklist included a number of issues such as availability of Isoniazid and pyridoxine, patient registers, presence of IPT guidelines, standard screening tool (TSQ), and Information Education and Communication (IEC) materials including fliers, pamphlets and posters from each selected health care facility.

For quantitative part of the study, the researchers conducted review of patients´ records using structured data collection form from IPT registries of each selected health care facility to generate information on the number of PLHIV registered in CTCs and provided with IPT between January 2015 and January 2017. In this study IPT coverage was determined as the percentage of PLHIV started on IPT among all PLHIV registered in CTCs eligible for IPT. The first author and trained research assistants collected data for this study. The administration of the healthcare facilities provided special room close to CTC for conducting in-depth-interviews. Based on their experiences of providing and taking IPT, health care providers and patient interviewees were asked about their views on facility and patient barriers in the implementation of IPT for PLHIV. Researchers conducted observation at the end of all interviews in each selected health care facility. The study obtained ethical approval from the Muhimbili University of Heath and Allied Sciences Research Ethics Committee. Permission to conduct the study was also obtained at regional, district and health facility levels in the study area. Individual informed verbal consent was obtained from all participants. They were informed about anonymity and confidentiality issues, and that they could withdraw from the study at any time they wished.

Quantitative data were analyzed using SPSS for windows version 20 statistical software. Data were checked for completeness and correctness through proofreading while entering the data into the software. Descriptive statistics (frequencies and percentage) were employed and data were visualized using tables and bar graphs. Thematic analysis was used to analyze qualitative data. Thematic analysis approach involves identifying, analyzing and reporting patterns (themes) within data [18]. Audio-recorded data from the in-depth interviews were transcribed verbatim and translated into English. Qualitative data verification for accuracy and completeness was done through reading and re-reading by the researchers to ensure all recorded information and variations were identified. After transcription, codes were developed by the researcher based on the original terms used, and were matched. The transcripts and notes were analyzed thematically by categorizing them in line with the specific objectives (facility and patient barriers in the implementation of Isoniazid preventive therapy for people living with HIV). The codes were presented, discussed and checked by the research team. Tentative categories and sub-categories were created from the clustered codes, and subsequently main themes emerged based on the patterns and relationships between the categories. Main themes were illustrated with representative quotations.

## Results

The quantitative findings showed that 2148 (87.3%) out of the 2460 PLHIV registered in the HIV clinics between January 2015 and January 2017 were eligible for IPT, however only 964 (44.9%) were provided with IPT. All health facilities included in this study started implementation of IPT in the years 2014 to 2016. The study findings revealed that there were disparities of IPT coverage between studied health care facilities. Two facilities had high IPT coverage of (87.6% and 57.0%) while three other facilities had a much lower coverage of (10.6%, 18.2% and 35.6%). The lowest IPT coverage in Songea municipality was observed in the health care facility that started IPT implementation late in 2016 compared with other health care facilities (Table 1).

**Table 1:** IPT start year and coverage at the 5 health care facilities in Songea municipality, Tanzania January 2015 - January 2017

Health Facility	IPT start year	Number of PLHIV registered in CTC	Number of PLHIV eligible for IPT	Total number of PLHIV provided IPT	IPT Coverage (%) *
**Facility 1**	2014	662	497	177	35.6
**Facility 2**	2014	426	391	71	18.2
**Facility 3**	2015	414	395	226	57.0
**Facility 4**	2016	391	348	37	10.6
**Facility 5**	2015	567	517	453	87.6
**TOTAL**		**2460**	**2148**	**964**	**44.9**

* IPT coverage was determined as the percentage of PLHIV provided IPT among all PLHIV registered in HIV care eligible for IPT.

Twenty-one participants participated in the qualitative study (Table 2). Of these, 13 were health care providers and 8 were CTC patients. The mean age (±SD) of health care providers was 48.9 (±9.9) years while that of CTC patients was 45.7 (±8.4). Eleven (84.6%) of health care providers had more than one year working experience in HIV care (Table 2). Seven (33.3%) of the participants had education ≤ standard seven (Table 2).

**Table 2:** demographic characteristics of inter viewed health care providers and patients at 5 HIV clinics in Songea municipality, Tanzania, 2017

Demographic characteristics of participants		Health care providers (N = 13) Patients (N = 8)	
		**N**	**%**
**Age (years)**	<30	2	9.5
	30-70	19	90.5
**Sex**	Female	9	42.9
	Male	12	57.1
**Profession**	Nurse	5	23.8
	Pharmacist	3	14.3
	Doctor	5	23.8
	CTC Patient	8	38.1
**Education Level**	≤ Standard seven	7	33.3
	Certificate	8	38.1
	Diploma	4	19.1
	Advance Diploma	2	9.5
**Work experience in HIV care (years)**	<1	2	15.4
**Work experience in HIV care (years)**	≥1	11	84.6

**Figure 1:**
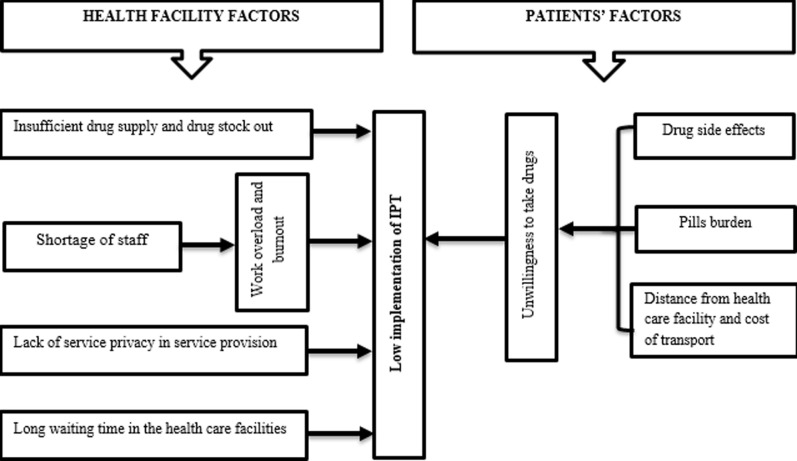
conceptual framework of barriers in the implementation of IPT for PLWHIV in Songea Municipality, Tanzania 2015-2017

**Health care facility barriers in IPT implementation**: on thematic analysis of the data, four themes (Figure 1) emerged showing institutional (health care facility) barriers in IPT implementation:

**Insufficient drug supply and drug stock out**: most interviewed health care providers reported the supply of Isoniazid and pyridoxine in Songea municipality is insufficient and irregular. They also said that sometimes they run out of stock. One of the respondents said that:

*“Isoniazid supply is a challenge, we provide IPT to a few clients. We check on our stock, and how many clients it can accommodate for six months, we take them as cohort. Therefore, every time we receive supply, we calculate it in that manner. We avoid providing IPT to every eligible client because it can lead to stock out which may result to creation of unnecessary drug resistance”* (R6H1-Doctor).

Interviewed CTC clients reported that they were upset by recurrent problem of insufficient supply and stock out of Isoniazid and pyridoxine in their CTCs. One of the respondents expressed that: *“We receive those drugs by groups, all patients don´t receive these drugs as required. When I received it, most of my fellow clients didn´t. They divide us into groups because they say drug supply is insufficient”* (R4H1-Patient).

**Shortage of staff**: most interviewed health care providers raised a concern about the number of available staff compared to CTC work load. One of the respondents said that: *“… hospital services have been expanded in our facility; staff have not been added as you know the number of staff decreases due to retirement, death and the government exercise of certificate verification for civil servants. All these results to burnout of the current providers which in turn leads to poor quality of service offered to clients because providers become tired”* (R12H4-Nurse).

Another respondent had this to say: *“I am a retired doctor; I was working here as soldier and a doctor. Due to shortage of staff they brought me back to provide support”* (R13H4-Doctor).

**Lack of service privacy**: the health care providers reported that the location of CTC building and the space available compromises with service privacy. One of the health care providers expressed that: *“… we don´t have special room for CTC although there is a building there, which is under renovation. We hope after shifting to that new building service privacy will be good. As you can see, we are two clinicians and sometimes we share one room which is not principally and ethically advised”* (R6H1-Doctor).

**Long waiting time**: interviewed patients reported long waiting time was a problem when they seek IPT services in the CTC. One of the respondents expressed that: *“We come here at 7:30 am but usually we stay until 9:00 am, and that is when they start providing services. In my opinions I request them to reduce waiting time and give us service on time so that we can proceed with other duties, some of our colleagues fail to come for this service due to this reason”* (R24H3-Patient).

**Patient barriers in IPT implementation**: study participants were asked to give their views about patient related barriers for IPT implementation. The analysis of the findings generated three main themes (Figure 1).

**Distance from health care facility and cost of transport**: most interviewed health care workers reported that distance from the health care facility and cost of transport were among the patient factors hindering IPT uptake in Songea municipality. One of the respondents expressed that: *“Some clients open a file here while they are from other districts, we usually counsel them during their first visit about distance and cost of transport but in some consecutive visits they miss their appointment dates. It is a challenge and we cannot force to refer them to health facilities close to their residences, they are free to choose where they wish to receive services”* (R7H1-Doctor).

Interviewed clients reported that in their area of residence there are dispensaries but do not have IPT service. One of the respondents said that: *“...I am living more than 10 kilometers away from this health center. I came here on foot because in some days I lack money to hire ‘boda-boda´ (motorcycle). I am trying to attend every appointment date though I am just pushing myself, it could be very helpful if the government could start providing this service to our nearby dispensary”* (R4H1-Patient).

**Drug side effects**: isoniazid side effects were reported by some interviewed health providers from clients they attended. One of the respondents expressed that: *“Some clients come with jaundice, rashes and neurotic symptoms, when we see that we stop and do investigation. When we agree that those side effects resulted from up taking Isoniazid, we stop giving them and they proceed with ART only”* (R6H1-Doctor).

When patients were asked about their experience of using Isoniazid and if it has any side effect, one patient reported to experience side effects of IPT: *“… I once came here and complained about itching and excessive urination especially during the night, the doctor told me to stop taking these drugs but I proceeded with ART”* (R20H5-Patient).

**Pill burden:** most health care providers reported that they prescribe drugs according to patient´s willingness. One of the respondents said that: *“Some clients say the number of drugs to take in a day is a lot, others refuse at all to take them, claiming that they have many drugs to take, they will get TB treatment if they get TB infection”* (R11H4-Pharmacist).

When patients were asked to provide their views about taking both TB and HIV drugs, most of them reported that they were taking two to three drugs in a day. One of the patients had said: *“The number of drugs in a day is not that large, am using two drugs in a day, ART and IPT, the problem is that we are supposed to take these drugs for life, we don´t have option because we are sick* (R3H1-Patient).

## Discussion

This study sought to understand the coverage of IPT implementation and explore facility and patient barriers in the implementation of IPT for PLHIV in Songea municipality, Tanzania. In this study IPT coverage was determined as the percentage of PLHIV provided IPT among all PLHIV registered in HIV care eligible for IPT. IPT coverage in Songea municipality was estimated to be 45% which is sub-optimal coverage since it is below the recommended WHO target level of 50%. IPT implementation in Songea municipality started in 2014 despite the fact that in Tanzania IPT was included in the national policy guideline for collaborative TB/HIV activities since 2008 [7]. The study showed disparities of IPT coverage between health care facilities; the regional referral hospital had IPT coverage of 87.6% because it received special priority from council and reginal health management teams in terms of supportive supervision, training and supply of the IPT. One health care facility that had the lowest coverage of 10.6% started IPT implementation late 2016 with insufficient supply of IPT. On average, this study reported low coverage of IPT (45%) in the study area. These findings are similar with other findings in Tigray region in Ethiopia where IPT coverage was reported to be only 20% [14]. Lack of IPT trainings prior to the introduction of IPT services was the main reason reported for low IPT implementation in Tigray region in Ethiopia [14].

**Health care facility barriers in IPT implementation**: the implementation of IPT service is mainly depending on the supply of Isoniazid and pyridoxine. Most health care providers and patients in this study reported that the supply of Isoniazid and pyridoxine was insufficient or irregular and sometimes run out of stock. This situation impedes the implementation of IPT service to PLHIV. Health care providers in Songea municipality have adapted a measure of checking on their stock and clients it can support for six months and take that as a cohort. This measure aims to avoid stock out and creation of unnecessary drug resistance. Shortage of Isoniazid and pyridoxine has also been reported in a study done in Ethiopia as a barrier hindering the implementation of IPT services in health care facilities [14]. The problem of shortage of supply of these drugs could arise at the level of quantification, procurement, and/or distribution. The stock out of pyridoxine in Songea municipality is an example of donor dependence problem. Pyridoxine and other opportunistic drugs were once supplied by a non-governmental organization operating in the southern part of Tanzania but it stopped supplying Pyridoxine and handle over this responsibility to the local government authorities. Since then, the supply of such drugs was no longer reliable, stock out of drugs became a recurrent challenge.

Most of the interviewed health care providers reported a shortage of staff caused by retirement, death, and the government exercise of certificate verification for civil servants. Shortage of staff in Songea municipality had an impact on IPT services implementation since the existing staff were overwhelmed with many activities and sometimes were forced to work overtime that resulted to staff burnout. Similar studies found that one of the barriers in the implementation of IPT for PLHIV was shortage of staff in some HIV clinics [19-21]. It was found that health care providers were inadequate and overwhelmed because of many other competing program activities that call for their daily attention [19-21]. Lack of service privacy in Songea municipality was caused by the location of the CTC building and the space available for service provision in the studied facilities. Lack of service privacy causes some clients to miss their drug collection dates. Some patients in the municipality were not willing to start IPT and others were not adhering to medication because of lack of service privacy in the health care facilities. A study on adherence to IPT in Ethiopia showed that respondents who don´t complain about service privacy were more likely to adhere to IPT than who complained. Service privacy was reported to greatly compromising an uptake of proper information, knowledge and behavior by the patient [22].

Long waiting time was found to impede implementation of IPT service in Songea municipality. Most patients reported that long waiting time when they seek CTC services reduce their time for doing day to day work for earning their income. Some clients decide not to start IPT because they fear to waste their time waiting for IPT service. Shortage of staff in CTCs is one of the reasons for long waiting time in Songea municipality. It is documented in other similar studies that long waiting time affects patient satisfaction and health service delivery, efficiency, quality, transparency, and accountability and also negatively impacts the outcome of ART treatment or any other program [23,24]. Long waiting time was regarded as a barrier for accessing ART services and the cause of high dropout rate in South Africa [24]. Similarly, shortage of medical staff and medical and laboratory supplies and lack of systematic appointment system are among the factors that partly contribute to long waiting time [25].

**Patient barriers in IPT implementation**: pills burden was reported by most interviewed participants as a barrier for the high uptake of IPT services in Songea municipality. The study participants were concerned with the number of drugs they take and the regime. Some patients were unwilling to add INH to ART and Cotrimoxazole due to fear of pills burden, that is, a minimum of 4 tablets per day for those who accepted IPT. Pills burden compromises with adherence and patient´s willingness to take drugs in Songea municipality. The problem of pills burden has also been reported by other similar studies [14,21], which showed that most providers indicated that they prescribed IPT based on the patients´ willingness to take drugs. They indicated that patients were unwilling to add INH to the ART and Cotrimoxazole due to fear of having many pills, a minimum of 10 tablets per day for those who accepted IPT [14,21,26].

Drug side effects is another barrier for IPT service implementation reported by both health care providers and patients. Most of the reported side effects due to use of INH were jaundice, dermatological and neurotic problems. Some of these side effects are known to be manageable but due to stock out of pyridoxine the prevalence of these problems is increased. A similar study from Ethiopia found related findings showing that side effects such as gastrointestinal irritation, peripheral neuropathy and hepatotoxicity were a barrier in IPT implementation. Moreover, they reported unavailability of pyridoxine in the health care facilities as the reason for failure to manage INH side effects [14]. Studies also show that most of these adverse effects make clients eligible for IPT to discontinue taking therapy [21,23,24].

Distance from health care facility and costs of transport were other barriers to IPT service implementation. Most interviewees reported that time wasted for travelling could be used for other productive work. Some patients who were living far away from health care facility and those who were poor were at risk of becoming non-compliant to medication and sometimes drug cessation. A Similar study done in Tanzania found that distance from the HIV clinic increased the risk of IPT cessation [27]. Another study done in UK found that non-compliant patients spent more time travelling to the treatment center than the compliant patients. The study also showed that of the non-compliant group, 55% indicated that they had not attended the clinics due to financial reasons [28].

## Conclusion

The study demonstrates that implementation of IPT in Songea municipality was low with only 45% of eligible PLHIV covered, which is lower than the WHO recommended IPT coverage of 50%. This low IPT coverage was a result of a number of barriers, which need attention of policy makers and implementers of IPT services. The government including local government authorities and managements of health care facilities, drug suppliers, and partners working in tuberculosis and HIV programs must be part of the solutions by ensuring that the identified health care facility and patient barriers are addressed in order to improve IPT uptake. In addition, service providers should work collaboratively with community leaders and community health workers to provide health education to PLHIV so as to create awareness about the benefits and importance of adherence to IPT medication regime and clinics appointments.

### What is known about this topic

- Globally, and Tanzania in particular, the implementation of Isoniazid Preventive Therapy for PLHIV is low despite the available evidence that it is efficacious;
- The low implementation of IPT is partly contributed by inadequate social support, stigma, distance from the health care facility, pills burden, forgetfulness and drug side effects, lack of IPT training, lack of supportive supervision, health care providers´ attitude towards IPT (fear of Isoniazid resistance), lack of good client-provider communication, and lack of patient registers.

### What this study adds

- Insufficient drug supply and stock out, shortage of staff, lack of service privacy, long waiting time and cost of transport hinder full scale implementation of Isoniazid preventive therapy;
- There is a need of local government authorities and managements of health care facilities, drug suppliers, and partners dealing with tuberculosis and HIV programs to collaboratively work together to improve the IPT uptake in the Southern part of Tanzania.
